# Extracellular Vesicles Derived from Primed Mesenchymal Stromal Cells Loaded on Biphasic Calcium Phosphate Biomaterial Exhibit Enhanced Macrophage Polarization

**DOI:** 10.3390/cells11030470

**Published:** 2022-01-29

**Authors:** Neha Rana, Salwa Suliman, Niyaz Al-Sharabi, Kamal Mustafa

**Affiliations:** Center for Translational Oral Research (TOR), Tissue Engineering Group, Department of Clinical Dentistry, Faculty of Medicine, University of Bergen, 5009 Bergen, Norway; neha.rana@uib.no (N.R.); Salwa.Suliman@uib.no (S.S.); N.Al-Sharabi@uib.no (N.A.-S.)

**Keywords:** mesenchymal stromal cells, biphasic calcium phosphate, biomaterial, extracellular vesicles or EV, monocytes, macrophages, immunomodulation, macrophage polarization, cytokines, secretome, bone regeneration

## Abstract

Mesenchymal stromal cells (MSC) loaded on biphasic calcium phosphate biomaterial (MSC + BCP) have been used as an advanced therapy medicinal product to treat complex maxillofacial bone defects in patients. Further, MSC-derived extracellular vesicles (EVs) are established vehicles of paracrine factors, supporting inter-cellular communication between MSC and other interacting cell types, such as monocytes/macrophages. However, the information about the immunomodulatory potential of EVs derived from MSC and biomaterial constructs (MSC + BCP:EV) and inflammatory primed constructs (MSCp + BCP:EV) are scarce. Hence, we isolated and characterized EVs from these different systems, and compared their cytokine contents with plastic-adherent MSC-derived EVs (MSC:EV). When EVs from all three MSC systems were added to the primary blood-derived macrophages in vitro, significantly higher numbers of M0 (naive) macrophages shifted to M2-like (anti-inflammatory) by MSCp + BCP:EV treatment. Further, this treatment led to enhanced switching of M1 polarized macrophages to M2 polarized, and conversely, M2 to M1, as evaluated by determining the M1/M2 ratios after treatment. The enhanced macrophage modulation by MSCp + BCP:EV was attributed to their higher immunomodulatory (TNFα, IL1β, IL5), angiogenic (VEGF), and chemokine-rich (RANTES, MCP1, MIP1β) cytokine cargo. In conclusion, we successfully isolated and characterized EVs from MSC + BCP constructs and demonstrated that, depending upon the tissue microenvironment, these EVs contribute towards modulating the macrophage-mediated inflammation and healing responses. The study offers new insights into the use of biomaterial-induced EVs for MSC secretome delivery, as a step towards future ‘cell-free’ bone regenerative therapies.

## 1. Introduction

The therapeutic potential of mesenchymal stromal cells (MSC), considering their multilineage differentiation potential and a unique secretome, is a promising tool for regenerative medicine [1]. One of the strategies in bone tissue engineering (BTE) involves transplantation of MSC along with bioactive synthetic materials to repair and regenerate bones at the defect sites [2]. A variety of synthetic bioceramic materials are used in BTE applications, but biphasic calcium phosphate (BCP) biomaterial consisting of 20% hydroxyapatite (HA) and 80% beta-tricalcium phosphate (β-TCP) has emerged as a preferred choice for MSC-mediated bone regeneration, both preclinically and clinically [3,4].

The pro-regenerative effects of transplanted MSC are shown to be related to their differentiation capacity into specialized cell types [5]. However, recent evidences suggests that MSCs rely on their active paracrine secretions, stimulating a variety of host cells (including immune cells), and the resulting multicellular crosstalk consequently impacts their regenerative potential [6,7]. Among innate immune cells, macrophages are identified as primary effector cells that orchestrate the inflammation and healing mechanisms during MSC-mediated regenerative processes [8,9]. The transplanted MSC are known to favor the switching of M1 polarized (classically activated) macrophages to M2 polarized (alternatively activated), which resolves tissue inflammation and promotes healing [9,10,11]. Further, the MSC secretome, at least partly, mediates the functional macrophage switch [12,13]. The MSC secretome harbors bioactive molecules (cytokines, chemokines, and growth factors) both in a free state (soluble fraction) as well as encapsulated into lipid-bilayered sub-cellular structures called extracellular vesicles (EV fraction) [14].

EVs are recognized as one of the most potent modes for transferring cellular information during regenerative processes, and could be in the form of proteins, lipids, mRNA, miRNA, and bioactive cytokines [1]. Further, they can be categorized into different subtypes based on their size or biogenesis mode, i.e., exosomes (30–150 nm), microvesicles (100–1000 nm) and apoptotic bodies (1000 nm–5 µm). However, in the absence of specific markers and overlapping sizes, the term ‘small EVs’ (sEV) applies to both exosomes and microvesicles up to 200 nm in size [15]. Recent evidence points towards MSC:sEV being critical in cellular communication [16,17,18]. In addition, modulating the in vitro culture conditions of MSC via hypoxic pretreatment [19] or by inflammatory stimulus [20,21] favorably alters their secretome [22,23], including EV composition [21], thus enhancing the paracrine properties of the cells. In a similar manner, the cytokine secretion profile of MSC was shown to be greatly impacted by the surface topographies of their in vitro microenvironments, leading to variable cell morphologies [24]. Hence, we hypothesized that MSC cultured with BCP granules could release a modified secretome (including EVs) capable of enhanced immunomodulation. Moreover, this enhancement could be involved both in terms of attenuation of pro-inflammatory response and promotion of anti-inflammatory response [25]. In the context of clinical maxillofacial bone augmentation procedures, when MSC mixed with BCP granules (MSC + BCP) are surgically transplanted into the defect sites, they promote the augmentation of mandibular bone [4]. The physical and chemical properties of biomaterials are known to influence their interaction with MSC and the release of osteogenic molecules in the secretome [26]. In line with this, we evaluated the in vitro osteogenic and healing response of MSC + BCP-derived conditioned media compared to MSC alone, and found that the former produced more pro-bone-regenerative secretome than the latter (unpublished manuscript). However, within this system, the contribution of the EVs derived from MSC + BCP-conditioned media, and additionally, the effect of cytokine priming on these EVs, still remains unknown.

In the current study, EVs derived from MSC + BCP constructs were evaluated for their role in regulating primary human macrophage-mediated inflammation. We isolated and characterized EVs derived from human bone marrow MSC under different conditions, that is, when MSC were cultured alone or with BCP biomaterial and with inflammatory cytokine priming (+TNFα and +IL1β). Further, maturation and polarization properties of peripheral blood-derived macrophage subtypes (M0, M1 and M2) were examined by treating them with each of these EV groups in vitro. Through this, we aimed to explore the EV-mediated paracrine macrophage interactions during clinical MSC + BCP transplantation, to help build advanced tissue-engineered EV (cell-free)-based regenerative strategies.

## 2. Materials and Methods

### 2.1. EV Isolation from MSC Conditioned Medium

Pre-characterized human bone marrow mesenchymal stromal cells (MSC) were used in this study [27,28]. MSC (passage 3) derived from three different individuals (Regional Ethical Committee reference number: 2019/7199/sør-øst C) were thawed from liquid N2 and cultured in Dulbecco’s Modified Eagle’s Medium (DMEM, Invitrogen, Carlsbad, CA, USA) supplemented with 1% penicillin/streptomycin (GE Healthcare, South Logan, UT, USA) and 10% fetal bovine serum (FBS; GE Healthcare). Thereafter, MSCs were trypsinized and washed thoroughly with 1× phosphate buffered saline (PBS, Invitrogen) to remove the traces of FBS. Cells were seeded at a density of 0.5 × 10^6^/mL with or without 25 mg of BCP biomaterial (MBCP+^®^, Biomatlante, Vigneux de Bretagne, France) in DMEM+ 1% penicillin/streptomycin (serum-free) in 96-well plates. The high seeding density (20 × 10^6^ cells per gram of BCP) used here was optimized previously in preclinical and clinical studies [4]. Additionally, MSCs were also primed (MSCp) by adding pro-inflammatory cytokines; recombinant human IL1β (10 ng/mL) and TNFα (10 ng/mL) (both from R&D Systems, Minneapolis, MN, USA) to the culture medium. Conditioned media (CM) was collected and pooled from 5 wells after 48 h of incubation at 37 °C and 5% CO_2_, from three different groups: MSC, MSC + BCP and MSCp + BCP. The pooled CM was centrifuged at 10,000× *g* for 10 min to remove cell debris and stored at −80 °C until further use.

### 2.2. Size Exclusion Chromatography

Collected CM from each of the three groups was passed through PURE-EV size exclusion chromatography (SEC) columns (HBM-PEV-5, HansaBioMed Life Sciences Ltd., Tallinn, Estonia) following the manufacturer’s protocol. Briefly, each column was first equilibrated by passing 30 mL of filtered PBS, followed by loading the CM sample (up to 2 mL) into the column. Next, fractions of 500 µL (volume) were collected by dropwise addition of PBS into the column. Eluted fractions, F1-F25, were collected for each sample and analyzed for nanoparticle size distribution by a dynamic light scattering (DLS) method using a Zetasizer Nano ZSP device (Malvern, UK). The protein concentration of each fraction was measured by Pierce^®^ Bicinchoninic Acid Protein Assay (BCA, Thermo Scientific) according to the manufacturer’s protocol. As SEC recovers EVs mixed with some amount of free proteins [15], care was taken to pool fractions with the minimum protein amount (70–90 µg/mL) and a lower size range to collect small EVs (mean size around 200 nm). Hence, fractions F8-15 were pooled and stored at −80 °C for downstream analysis.

### 2.3. Transmission Electron Microscopy

The intact EVs (10 µg resuspended in PBS) were fixed with 2% glutaraldehyde (1:1) for 30 min. A drop of fixed EVs was placed on parafilm, and a formvar carbon coated copper grid (200 mesh) was gently positioned over this drop for 10 min. Excess sample was removed with blotting paper, and the grids (along with the loaded EVs) were sequentially washed twice with 100 μL milli-Q water, by brief contact. Next, the EV coated grids were stained with 1.5% uranyl acetate for 12 s. Thereafter, they were positioned on a paper with the coated side up and air dried for 5 min. The preparations were examined with a JEOL JEM1400 transmission electron microscope (JEOL Ltd., Tokyo, Japan).

### 2.4. Cytokine Bioplex Immunoassay

A human cytokine 27-plex assay (Table 1) was used with a Bio-Plex^®^ 200 System (both from Bio-Rad Laboratories, CA, USA) to measure the cytokine content of EVs isolated from human MSC (*n* = 2) including all experimental groups (as per the manufacturer’s instructions). Before the assay, EV samples were lysed by using triton X-100 in a 1% final concentration to allow the release of encapsulated cytokines [14]. The protein concentration of the lysed EVs was measured by BCA assay following the manufacturer’s protocol. Individual cytokine concentrations (pg/mL) obtained thereafter were normalized to the total protein concentration (µg/mL) for each sample, and the results were presented as the pg/µg of total protein.

### 2.5. CD14+CD16- Human Monocyte Isolation from Peripheral Blood

Peripheral blood mononuclear cells (PBMC) were isolated from healthy donor buffy coat preparations (Dok-ID AIT-55879, provided by the Blood Bank, Haukeland University Hospital, Bergen, Norway) by using Ficoll Paque PLUS (GE Healthcare, Little Chalfont, UK). Briefly, buffy coats were diluted with sterile PBS in a 1:1 ratio and layered over Ficoll Paque PLUS in a ratio of 4:3 (4 parts blood and 3 parts Ficoll). The PBMC layer was separated after gradient centrifugation at room temperature (760× *g*, 20 min, brakes off). The separated cell layer was subjected to a total of four washes with 10 mL of PBS per wash (350× *g*, 10 min, brakes on). The final cell pellet (PBMC) was re-suspended in a buffer containing PBS, 0.5% BSA, and 2 mM EDTA solution and cells were counted.

CD14+CD16- monocytes were separated from PBMC by magnetically activated cell sorting (MACS) using classical monocyte isolation kit (MACS, Miltenyi Biotec GmBH, Bergisch Gladbach, Germany), according to the manufacturer’s protocol. Unlabeled monocytes recovered in the flow-through were counted and seeded for the experiments.

### 2.6. Generation of M0/M1/M2 Differentiated Macrophages and EV Treatment

Isolated monocytes were seeded into 48-well plates at a density of 2 × 10^5^ cells/cm^2^ in Roswell Park Memorial Institute 1640 medium (RPMI, Thermofisher Scientific, Oslo, Norway) supplemented with 10% FBS (heat inactivated) and 20 ng of macrophage colony stimulating factor (MCSF) at 37 °C and 5% CO_2_. They were allowed to differentiate in MCSF-containing media for the next 6 days, replacing with fresh media every 3rd day. At day 6, the adherent macrophage population (M0) was characterized for the expression of surface antigens CD14, CD16 and CD68 using flow cytometry. To assess the effect of EVs on the maturation of macrophages, the cells were treated with serum-free medium for 24 h of incubation with the following groups: (1) MSC:EV, (2) MSC + BCP:EV, (3) MSCp + BCP:EV. Cells incubated with an equal volume of PBS served as controls (no-EV control).

For obtaining M1/M2 polarized macrophages, the culture media was supplemented with 50 ng/mL of lipopolysaccharide (LPS) (for classically activated macrophages, M1) or interleukin 4 (IL4) (for alternatively activated macrophages, M2) and cells were incubated for 24 h. Afterwards, to assess the effect of EVs obtained from different groups in regulating macrophage polarization switch, EVs (5 µg/mL) were added separately to both M1 and M2 cultures and incubated for an additional 24 h.

### 2.7. Uptake of EVs by Macrophages

To evaluate the EV uptake/internalization by macrophages, 5 µg of EVs (per group) were fluorescently labeled with a green lipophilic dye, PKH67 (Sigma-Aldrich, St. Louis, MO, USA) following the manufacturer’s protocol. To remove excess dye, samples were washed with 1% BSA. Next, they were transferred to 300-kDa MWCO filters (Vivaspin 20, Sartorius Stedim Biotech GmbH) and centrifuged at 4000× *g* for 3 min at 4 °C. A control sample was processed in parallel containing equal volume of 1xPBS. The EVs from each group were labelled, and the controls were mixed with RPMI media (serum-free) and added onto the macrophages cultured in an 8-well μ-slide (Ibidi GmbH, Germany), for 24 h at 37 °C and at 5% CO_2_. The cultures were then washed twice with PBS, before they were incubated with a CFSE cell tracker cytoplasmic stain (CellTracker™ Red CMTPX dye, Thermo Fischer) for 15 min, followed by fixation with 4% paraformaldehyde solution (PFA) for 20 min. To detect nuclear DNA, cells were stained with a 4′,6-diamidino-2-phenylindole (DAPI) stain for an additional 20 min. Cellular uptake of EVs was immediately observed and recorded using a confocal laser microscope (TCS SP8; Leica, Germany).

Quantification of the relative number of EVs internalized by macrophages was performed by calculating corrected total cell fluorescence (CTCF) for approximately 40 macrophages (per group) using Image J software (v1.52a, National Institute of Health, Bethesda, MD, USA). Briefly, area and integrated fluorescence intensity per cell (in PKH67 stained/green channel) was determined after normalizing for color threshold and adjusting for background fluorescence (via the analyze/measure function). The CTCF was calculated by using the formula; CTCF = Integrated Density − (Area of selected cell × Mean fluorescence of background readings) [29].

### 2.8. Flow Cytometry

Isolated EVs from different MSC groups were analyzed for EV-specific proteins by immuno-affinity-based Dynabeads^®^ magnetic separation technology following the manufacturer’s protocol (CD63-10606D and CD81-10622D, Thermo Fischer Scientific, Norway). Briefly, 100 µL of the EV sample (around 5 µg of protein) was incubated with 20 µL of each of the two Dynabeads (CD63 and CD81), respectively, and incubated overnight (18–22 h) at 4 °C on a rotator. The next day, bead-bound EVs were washed (using a magnetic stand) and incubated with 20 µL of each antibody for 60 min on a sample shaker (room temperature). Stained bead-bound EVs, along with isotype controls, were washed, and data were acquired using a BD Accuri C6 flow cytometer (BD Biosciences, San Jose, CA, USA).

For studying the effects of EV treatment (MSC/MSC + BCP/MSCp + BCP) on the maturation and potential skewing of human M0 macrophages into M1 or M2 sub-phenotypes, cells were stained with the following fluorescent tagged anti- human monoclonal antibodies; APC CD14, FITC CD16, PE CD68, APC CD80, FITC CD206, FITC CD163 (all from Biolegend, San Diego, CA, USA) and APC CD86 (from BD Biosciences, USA). Next, to study the effects of EVs on M1 and M2 polarized macrophages individually, M1 and M2 macrophages were stained separately with antibodies (CD80, CD86, CD206 and CD163). For all of the experiments, cells were blocked with Fc block (Human TruStain FcX™, Biolegend) used at 1:10 dilution for 20 min at 4 °C, followed by staining with antibodies at a 1:200 dilution (each) for 30 min at 4 °C. The stained samples, along with their unstained controls and the isotype control (PE Mouse IgG1, κ, BD Biosciences), were acquired on a BD Accuri C6 flow cytometer. Data were analyzed using flow cytometry software (FlowJo V10.6.2, Flowjo, LLC, Ashland, OR, USA).

### 2.9. Gene Expression Analysis

Gene expression for M1, M2 and other functional macrophage-related genes (all from TaqMan^®^ real-time PCR assays, Thermo Fisher Scientific, Norway) were used to access the macrophage maturation state after EV treatment using a quantitative real-time polymerase chain reaction (qPCR) (Table 2). Briefly, 200 ng of RNA was reverse transcribed into cDNA using a high-capacity complementary DNA reverse transcription kit (Applied Biosystems, CA, USA). The expression levels of target genes were normalized relative to the housekeeping gene, glyceraldehyde 3-phosphate dehydrogenase (GAPDH). The data were analyzed by the ΔΔCT method and the results were presented as fold changes (linear) in each of the EV-treated experimental groups relative to the untreated control (*n* = 3).

### 2.10. Statistical Analysis

Statistical analyses were performed using the Prism 9.0 software (GraphPad Software, San Diego, CA, USA). Data were presented as means (+SD), unless specified. Both flow cytometry and qPCR data were analyzed using a two-way ANOVA with Tukey’s multiple comparison tests. *p* < 0.05 was considered statistically significant.

## 3. Results

### 3.1. Isolation and Characterization of MSC and MSC + BCP Derived EVs

**Figure 1:**
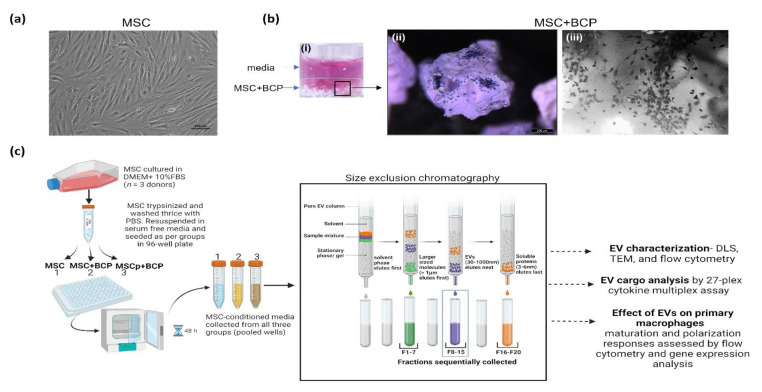
Isolation of EVs from bone marrow mesenchymal stromal cell (MSC) conditioned media: (**a**) representative phase-contrast microscope image of plastic-adherent MSC (scale bar = 100 µm); (**b**) (i) experimental set-up showing MSC cultured on top of BCP granules (white). MSC adhered on surface of BCP granules are shown after crystal violet staining (blue) via representative (ii) stereomicroscope image and (iii) phase contrast microscope image. Scale bars = 200 µm and 100 µm, respectively; (**c**) graphical schematics of steps involved in preparing conditioned samples (CM) and EV isolation by size exclusion chromatography (SEC). Briefly, CM was collected from around 2.5 × 10^6^ MSC/group, cultured in serum free media for 24 h. CM (0.5 mL/per group) was passed through matrix-filled SEC columns (Pure EV, hansa biomed). Fractions of 500 µL each were sequentially eluted using PBS (as depicted). Fractions 8–15 were pooled and used for experimental analysis. Graphical schematics shown here is created with BioRender.com.

MSCs cultured alone or in combination with BCP granules displayed typical spindle-shaped fibroblastic morphology (Figure 1a,b(iii)). In addition, both primed (MSCp + BCP) and unprimed (MSC + BCP) cells, cultured on BCP granules, showed a colony-like growth pattern, as observed by stereomicroscope 3D image (Figure 1b(ii),). MSCs from three different individuals were cultured and the CM was collected from each group (MSC, MSC + BCP and MSCp + BCP). Further, the CM was utilized to isolate EVs by size exclusion chromatography (SEC). Graphical illustration of the steps involved in this process are shown (Figure 1c).

**Figure 2:**
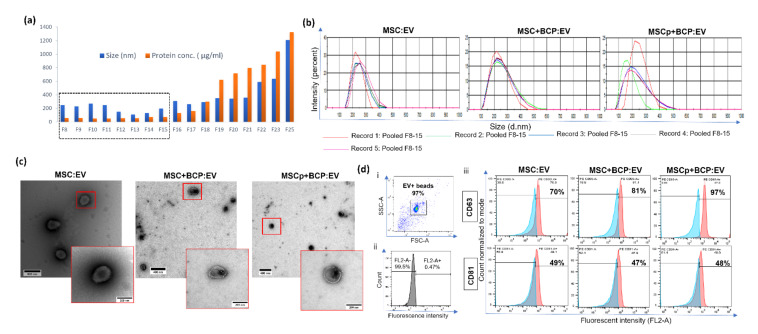
Characterization of EVs: (**a**) Representative graph showing size of eluted EV fractions (F8-F25) versus protein concentrations measured via BCA assay. Highlighted F8-F15 were used for experimental analysis; (**b**) representative size distribution graphs by dynamic light scatter (DLS) of selected pooled fractions (F8-F15) for each experimental group—MSC:EV, MSCp:EV, MSC + BCP:EV & MSCp + BCP:EV—showing that EVs in each group have a mean range of particle size of 150–250 nm; (**c**) transmission electron microscopy images of pooled EV fractions for MSC:EV, MSC + BCP:EV & MSCp + BCP:EV. Scale bars (left to right): 500 nm, 400 nm and 400 nm, respectively. Scale bar for inset images; 200 nm, for all. (**d**) Representative flow cytometry analysis of EV-associated transmembrane proteins, CD63 and CD81. (i) Side scatter area vs. forward scatter area plot showing gated EV+ beads population; (ii) isotype control–PE Mouse IgG1; (iii) histograms showing fluorescent intensity for PE-CD63 and PE-CD81 across experimental EV groups. Blue—isotype control, Red—stained EV+ beads.

EVs from collected SEC fractions (8–25) were compared for their particle size and protein concentrations (Figure 2a). Pooled fractions F8-15 for each of the EV groups were comparable and revealed a mean particle size in the range of 150–250 nm (Figure 2b). Nevertheless for each MSC group, the observed mean size ranges were as follows: MSC:EV, 180–250 nm; MSC + BCP:EV, 150–250 nm; and MSCp + BCP:EV, 120–270 nm. No significant differences were found in the size or protein amount of the EVs derived from plastic-adherent and BCP-adherent MSC-derived EVs. Next, the EVs were visualized using transmission electron microscopy. All of the EV groups showed similar round, cup-like morphology, with the double membrane structure being clearly visible in the MSC + BCP:EV (Figure 2c). Further, EV-associated transmembrane proteins CD63 and CD81 (tetraspanins) were evaluated via bead-based flow cytometry analysis. All three EV groups showed considerable CD63 and CD81 expression. Interestingly, MSCp + BCP showed the highest amount of CD63 expression (97%) as compared to EVs from unprimed MSC and MSC + BCP, while CD81 expression was found to be relatively constant across all three experimental groups (47–49%) (Figure 2d).

### 3.2. EV Cytokine Bioplex Immunoassay

**Figure 3:**
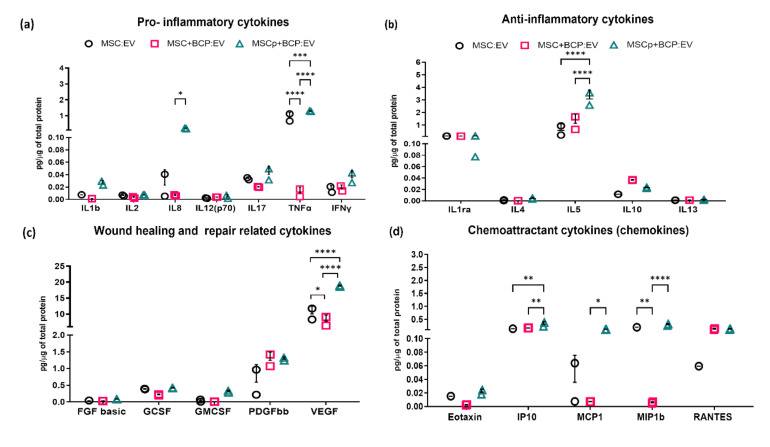
Cytokine analysis EV cargos by bioplex assay: (**a**) pro-inflammatory cytokines; (**b**) anti-inflammatory cytokines; (**c**) wound healing and repair-related cytokines; (**d**) chemoattractant cytokines. Concentration of each analyte (pg/mL) was normalized to total protein concentration of that sample (µg/mL). Normalized concentrations and standard deviations are shown in Table 3. Statistical analyses are based on 2-way ANOVA model with Tukey’s multiple comparison tests; * *p* < 0.05; ** *p* < 0.001; *** *p* = 0.0001; **** *p* < 0.0001.

The encapsulated cytokines in the EV compartment were evaluated by human cytokine 27-plex assay. Those detected in an observable concentration range (22 out of 27) were categorized based on their functional relevance (Table 3). Among the pro-inflammatory cytokines, the lowest amounts were detected in MSC + BCP:EV, as compared to both MSC:EV and MSCp + BCP:EV. Noticeably, the IL8 cytokine level was significantly increased in MSCp + BCP:EV, as compared to MSC + BCP:EV (*p* < 0.05). Additionally, MSCp + BCP:EV expressed significantly more TNFα level as compared to both MSC:EV (*p* = 0.0001), and MSC + BCP:EV (*p* < 0.0001) (Figure 3a). Evidently, the inflammatory priming of cells was reflected in their EV fractions. In the case of anti-inflammatory cytokines, the IL10 concentration was slightly greater in MSC + BCP:EV, and IL5 was significantly greater in MSCp + BCP:EV as compared to both MSC:EV and MSC + BCP:EV (both *p* < 0.0001). Other anti-inflammatory cytokine levels (IL1rα, IL4, IL15) were comparable among the EV groups (Figure 3b). In the case of wound healing and repair-related cytokines, VEGF levels were hampered due to the presence of BCP in MSC + BCP:EV but priming seemed to have rescued this effect. Consequently, it was significantly greater in both EVs derived from MSC without BCP (MSC:EV) (*p* < 0.05) and primed MSC + BCP (MSCp + BCP:EV) (*p* < 0.0001) (Figure 3c). Similarly, all of the chemokines, i.e., IP10 (CXCL10), MCP1 (CCL2) and MIP1b (CCL4), were significantly greater in MSCp + BCP:EV. Interestingly, although not statistically significant, RANTES (CCL5), a chemokine family expressed by osteoblasts [30], was present in a considerably greater quantity in EVs derived from both MSC + BCP groups, in comparison to EVs derived from MSC alone (Figure 3d).

### 3.3. Uptake of EVs Derived from Different MSC Groups by Macrophages in Culture

**Figure 4:**
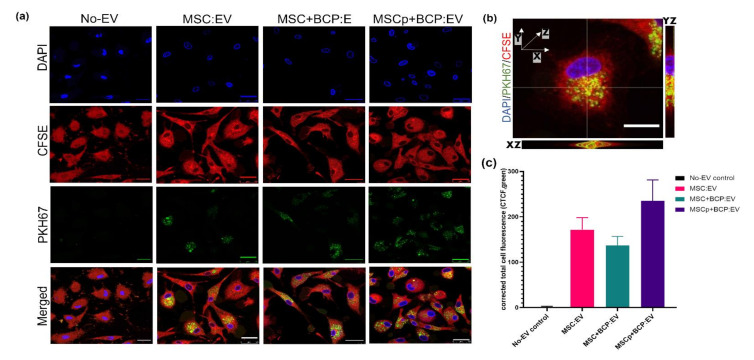
EVs secreted from all groups are internalized by primary macrophages in culture: (**a**) representative confocal photomicrographs showing EVs from all experimental groups being internalized by the macrophages after 24 h, but not in no-EV control (with 1xPBS stained with PKH67 dye). Scale bar = 25 µm; (**b**) orthogonal view of single stained macrophage after stacked Z projection showing EVs being internalized (yellow-merged) in both YZ (vertical) and XZ (horizontal) planes. Scale bar = 10 µm; (**c**) quantification of CTCF/mean integrated fluorescence density in green channel for all groups (*n* = 40 cells). Blue—DAPI-stained nuclei, Red—CFSE-stained cytoplasm, Green—PKH67-stained EVs.

The PBMC-derived CD14+CD16- monocytes were differentiated into macrophages for 6 days using MCSF. Afterwards, the cells were characterized for monocyte/macrophage cell surface markers (Supplementary Figure S1). The EV uptake by macrophages was evaluated by treating the fluorescently labelled EVs (PKH67 dye, green) with the macrophages in vitro, and uptake was visualized after 24 h of culture (Figure 4a). 3D projection suggests that EVs were present inside the cells and not just on the surface. Orthogonal views of same cell are shown (Figure 4b). The uptake or internalization efficiency of EVs obtained from different MSC groups was compared by quantifying the mean integrated fluorescence intensity in the green channel. It appeared that MSCp + BCP:EVs have enhanced potential to be internalized by macrophages, although no statistically significant difference was found in the fluorescence intensities between different EV groups (Figure 4c).

### 3.4. Effect of MSC-Derived EVs on Macrophage (M0) Maturation

**Figure 5:**
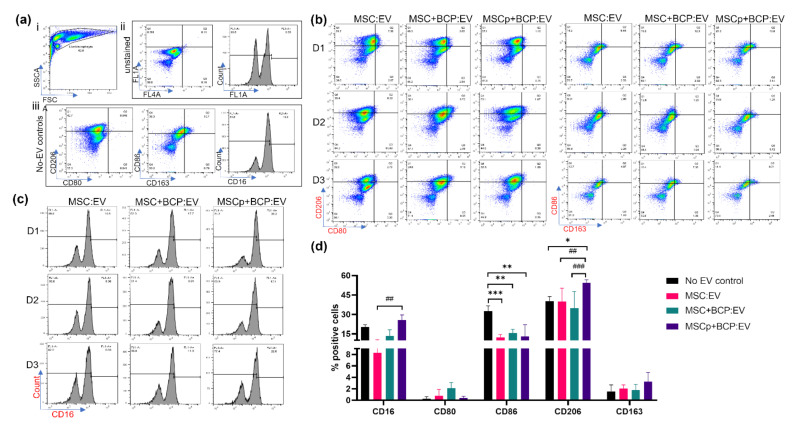
Effect of MSC-EV treatment on macrophage maturation and polarization: (**a**) (i) Side scatter vs. forward scatter plot showing live gated population of macrophages used for analysis. Dot plots or histogram showing expression of markers in (ii) unstained cells and (iii) no-EV controls. Expression in unstained cells was used for gating; (**b**) representative biaxial dot plots showing expression of CD206/CD80- and CD86/CD163-stained cells for each of three MSC donors (D1,D2,D3) and experimental groups (MSC:EV/MSC + BCP:EV/MSCp + BCP:EV) after flow cytometry; (**c**) representative histograms showing CD16 expression among three MSC donors and experimental groups; (**d**) quantitative mean expression from all three donors for percentage-positive expression of each marker across EV groups and no-EV controls (*n* = 3 per donor). Statistical analyses are based on 2-way ANOVA model with Tukey’s multiple comparison tests. (*/#) * *p* < 0.05; ** *p* < 0.001; *** *p* = 0.0001; (* represents *p* value of control w.r.t groups, i.e., MSC:EV/MSC + BCP:EV/MSCp + BCP:EV; # represents *p* value among the groups, i.e., MSC:EV w.r.t MSC + BCP:EV or MSCp + BCP:EV, or vice-versa).

The EVs were added to naïve (M0) macrophages for 24 h. After EV treatment, macrophages were evaluated for change in their M1 (CD80, CD86) and M2 (CD206, CD163) specific markers, and also for CD16 expression, via flow cytometry. Unstained cells were used to set gates for both the treated and untreated controls (Figure 5a). Representative dot plots or histograms for each of the three MSC donors with each marker are shown (Figure 5b,c). Quantifying the mean expression from all EV preparations revealed that CD86 (M1) expression was significantly downregulated in macrophages treated with MSC:EV, MSC + BCP:EV and MSCp + BCP:EV compared with the no-EV control (*p* = 0.0001, *p* < 0.001 and *p* < 0.001, respectively). In contrast, CD206 (M2) expression was significantly upregulated only in MSCp + BCP:EV-treated macrophages compared with a no-EV control (*p* < 0.05). Interestingly, CD206 expression in MSCp + BCP:EV-treated macrophages was also significantly upregulated as compared with macrophages treated with MSC:EV and MSC + BCP:EV (*p* < 0.001 and *p* = 0.0001, respectively). However, CD80 and CD163 were found to be overall less expressed on macrophages, and their expression did not show any significant changes within the EV-treated groups or compared with the no-EV control. Additionally, macrophage maturation related marker CD16 expression was significantly upregulated in macrophages treated with MSCp + BCP:EV, compared with MSC:EV-treated macrophages (*p* < 0.001).

### 3.5. Effect of MSC-Derived EVs in Regulating Macrophage Gene Expression

**Figure 6:**
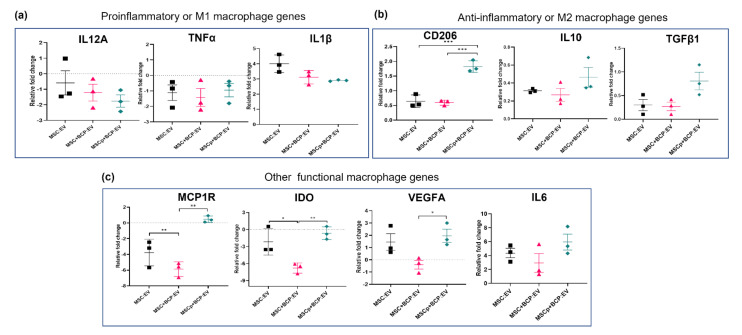
Effect of MSC-derived EVs in regulating macrophage gene expression: scatter plots showing relative fold change (mean with SEM) after treatment with MSC-derived EVs from 3 donors in respective groups; (**a**) M1 macrophage specific genes (IL12A, 1L1β and TNFα); (**b**) M2 macrophage specific genes (CD206, IL10, TGFβ1); (**c**) other functionally relevant macrophage genes (MCP1R, IDO, VEGFA, IL6). Relative linear expression shown is based on ∆∆CT values obtained after normalizing with no-EV control expression. Statistical analyses are based on 2-way ANOVA model with Tukey’s multiple comparison tests; * *p* < 0.05; ** *p* < 0.001; *** *p* = 0.0001.

Gene expression was studied after treating macrophages (M0) with different EV groups and normalizing the expression with untreated macrophages (no-EV control). The macrophage genes included were categorized as pro-inflammatory (M1 subtype specific), anti-inflammatory (M2-subtype specific), or other functionally relevant genes (Table 2). It was found that, among the pro-inflammatory genes, the expression of IL12A and TNFα was downregulated, with non-significant (ns) variation within the EV groups. However, the IL1β gene was upregulated, with more expression in MSC:EV-treated cells and around a 1.5-fold reduced expression in cells treated with MSC + BCP:EV and MSCp + BCP:EV (ns) (Figure 6a). Among the anti-inflammatory genes, CD206 expression was found to be significantly upregulated in MSCp + BCP:EV-treated cells compared to cells treated with MSC:EV (*p* < 0.0001) and MSC + BCP:EV (*p* < 0.0001). Similarly, IL10 and TGFβ1 were also upregulated in MSCp + BCP:EV-treated cell (ns) (Figure 6b). In case of other functionally relevant macrophage genes, we found that both IDO and MCP1R (CCR2) genes were significantly more downregulated by MSC + BCP:EV treatment, compared to MSC:EV (*p* < 0.001). Further, in case of other genes, also regulating macrophage functions, MCP1R was upregulated only in cells treated with MSCp + BCP:EV (*p* < 0.001) vs. MSC + BCP:EV, and IDO was significantly more downregulated in MSC + BCP:EV-treated cells as compared to cells treated with MSC:EV (*p* < 0.05) and MSCp + BCP:EV (*p* < 0.001). Similarly, VEGFA and IL6 showed upregulation in macrophages upon EV treatment, with lower expression of these genes in MSC + BCP:EV-treated cells (significant in VEGFA, *p* < 0.05), compared to that in MSCp + BCP:EV-treated cells (Figure 6c).

### 3.6. Effect of MSC-Derived EVs on M1 Polarized Macrophages

**Figure 7:**
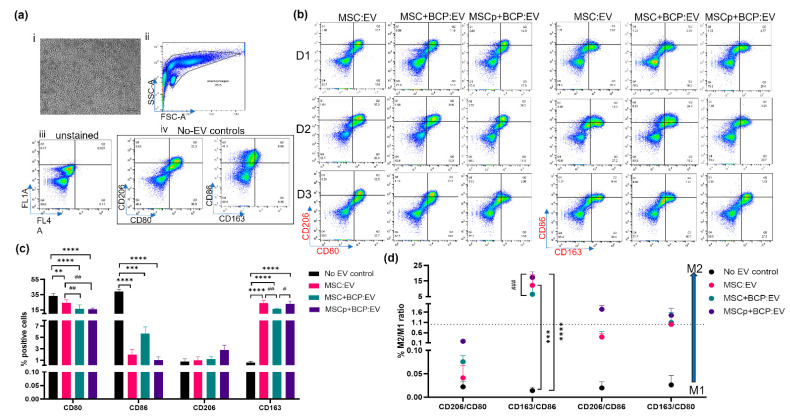
Effect of MSC-derived EVs on M1 polarized macrophages: (**a**) (i) light microscope image showing M1 macrophage morphology (ii) side scatter vs. forward scatter plot showing live gated population of macrophages used for analysis. Dot plots showing expression of markers in (iii) unstained cells and (iv) no-EV controls. Expression in unstained cells was used for gating; (**b**) representative biaxial dot plots showing expression of CD206/CD80- and CD86/CD163-stained cells for each of the three MSC donors (D1,D2,D3) and experimental groups (MSC:EV/MSC + BCP:EV/MSCp + BCP:EV) after flow cytometry; (**c**) quantitative mean expression from all three donors for percentage positive expression of each marker across EV groups and no-EV controls (*n* = 3 per donor and per group); (**d**) mean M2/M1 ratios obtained for each of the M2 markers (CD206, CD163) and M1 markers (CD80, CD86). Dotted line indicates ratio = 1; statistical analyses are based on 2-way ANOVA model with Tukey’s multiple comparison tests. (*/#) ** *p* < 0.001; *** *p* = 0.0001; **** *p* < 0.0001 (* represents *p* value of control w.r.t groups, i.e., MSC:EV/MSC + BCP:EV/MSCp + BCP:EV; # represents *p* value among the groups, i.e., MSC:EV w.r.t MSC + BCP:EV or MSCp + BCP:EV, or vice-versa).

Macrophages were polarized into classically activated (M1) or pro-inflammatory type by incubating them with LPS-supplemented (50 ng/mL) culture media for 24 h. The resulting M1 macrophages showed more cells with typical short and rounded morphologies and fewer elongated ones, in accordance with the previous report [31] (Figure 7a(i)). These cells also showed high expression of the M1 macrophage-associated costimulatory molecules (CD81 and CD86), and lower expression of the M2 macrophage-associated mannose receptor (CD206), as wells as the scavenger receptor (CD163) (no-EV control, Figure 7a(iv),c).

The EVs from the three MSC donors and each experimental group were incubated with the M1 macrophages for 24 h (Figure 7b). After treatment, it was found that the mean expression of both CD81 and CD80 was significantly decreased, while that of CD163 was significantly increased in M1 macrophages, as compared to the control (Figure 7c). Notably, CD80 was also significantly reduced among the EV groups, in cells treated with MSC + BCP:EV (*p* < 0.001) and MSCp + BCP:EV (*p* < 0.05), as compared to MSC:EV-treated cells. CD86 did not show a statistically significant variation among the EV groups, but the biggest decrease was seen in cells treated with MSC:EV and MSCp + BCP:EV. Further, CD163 was highly significantly expressed in all cells treated with MSC:EV, MSC + BCP:EV and MSCp + BCP:EV (*p* = 0.0001 to *p* < 0.0001). However, CD206 expression showed no statistically significant increase upon EV treatment, although it was at least 2.5-times higher in MSCp + BCP:EV-treated cells (Figure 7c).

Further, we assessed the percentage M2/M1 ratio (switch towards M2) using the positive ratio for each of the analyzed surface markers. It was found that for all four M2/M1 pairs analyzed, MSCp + BCP:EV-treated macrophages had a higher percentage of M2-switched cells, with CD163/CD86, CD206/86 and CD163/CD80 ratios being numerically more than one. Further, the ratio of CD163/CD86 was significantly higher after MSC:EV (*p* = 0.0001) and MSCp + BCP:EV (*p* < 0.0001) treatment as compared to the control. Among the groups, CD163/86 was significantly higher after MSCp + BCP:EV (*p* = 0.0001) treatment compared to MSC + BCP:EV, showing the greater impact of priming in comparison to the BCP biomaterial on EV-mediated macrophage polarization (Figure 7d).

### 3.7. Effect of MSC-Derived EVs on M2 Polarized Macrophages

**Figure 8:**
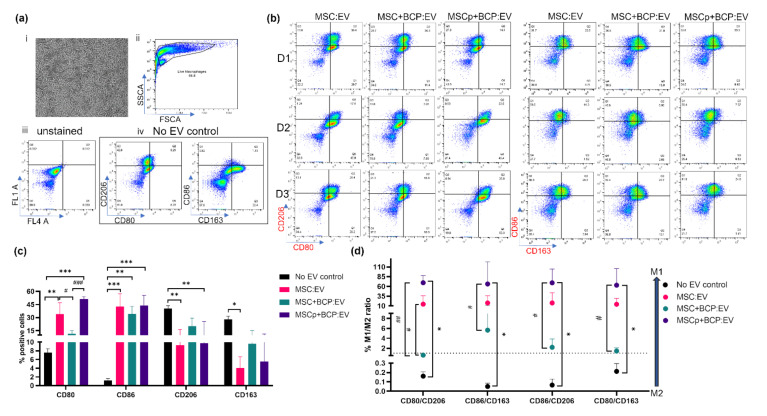
Effect of MSC-derived EVs on M2 polarized macrophages: (**a**) (i) light microscope image showing M2 macrophage morphology; (ii) side scatter vs. forward scatter plot showing live gated population of macrophages used for analysis. Dot plots showing expression of markers in (iii) unstained cells and (iv) no-EV controls. Expression in unstained cells was used for manual gating; (**b**) representative biaxial dot plots showing expression of CD206/CD80 and CD86/CD163 stained cells for each of three MSC donors (D1,D2,D3) and experimental groups (MSC:EV/MSC + BCP:EV/MSCp + BCP:EV) after flow cytometry; (**c**) quantitative mean expression from all three donors for percentage positive expression of each marker across EV groups and no-EV controls (*n* = 3 per donor and group) (**d**) Mean M1/M2 ratio obtained for each of the M1 markers (CD80,CD86) and M2 markers (CD206,CD163). Dotted line indicates ratios = 1. Statistical analyses are based on 2-way ANOVA model with Tukey’s multiple comparison tests. (*/#) * *p* < 0.05; ** *p* < 0.001; *** *p* = 0.0001; (* represents *p* value of control w.r.t groups, i.e., MSC:EV/MSC + BCP:EV/MSCp + BCP:EV; # represents *p* value among the groups, i.e., MSC:EV w.r.t MSC + BCP:EV or MSCp + BCP:EV, or vice-versa).

Macrophages were polarized into an alternatively activated subtype (M2-like) by incubating them with IL4-supplemented (50 ng/mL) culture media for 24 h. The resulting M2 polarized macrophages showed more cells with an elongated spindle shaped morphology, and fewer round ones [31] (Figure 8a(i)). These cells were also highly positive for the M2-like macrophage markers CD206 and CD163, and less positive for the M1 macrophage markers CD80 and CD86 (no-EV control; Figure 8a(iv),c).

The EVs from each experimental group were incubated with the M2 macrophages for 24 h (Figure 8b). After treatment, it was found that the mean expression of both CD80 (in MSC:EV and MSCp + BCP:EV) and CD86 (in all three groups) was significantly increased, whereas the expression of CD206 (in MSC:EV and MSCp + BCP:EV) and CD163 (in MSC:EV) was significantly decreased as compared to the control (Figure 8c). Among the EV groups CD80 was significantly less expressed in MSC + BCP:EV compared with MSC:EV (*p* < 0.05), and significantly less expressed in MSCp + BCP:EV compared with MSC + BCP:EV (*p* < 0.001). No statistically significant variations were found among the groups for other markers, i.e., CD86, CD206 and CD163 (Figure 8c).

Further, upon assessing the M1/M2 ratio (switch towards M1), it was found that MSCp + BCP:EV-treated macrophages had a significantly higher M1/M2 ratio compared to the control. This also applied to comparisons among the EV groups for all of the probable pairs: CD80/CD206, CD86/CD163, CD86/206 and CD80/CD163 (Figure 8d). Interestingly, the overall trend indicated that the smallest M1/M2 ratio was in MSC + BCP:EV treated cells, thus showing their resistance in mediating the reverse (M2 to M1) macrophage switch.

## 4. Discussion

Mesenchymal stromal cell (MSC)-derived extracellular vesicles (EVs) have emerged as a novel therapeutic tool due to their ability to promote paracrine communication, which is imperative to several MSC functions such as immune-modulation and angiogenesis [1,32]. Further, transplantation of autologous MSC, combined with biphasic calcium phosphate biomaterial (MSC + BCP), forms the current most promising tissue engineered clinical strategy to repair and regenerate lost bones [4,33]. In such treatment procedures, bone regeneration, and healing outcomes are known to be greatly impacted by MSC-immune cell interactions, and primarily by MSC-macrophage cross-talk [19,34]. Hence, in this study, we isolated extracellular vesicles from an MSC + BCP construct, and evaluated their role in active immune modulation with macrophages, regulating inflammation or healing during the early phase (24 h). It was found that EVs isolated from the primed MSC + BCP construct (MSCp + BCP:EV) have an increased potential to induce unpolarized/naive macrophages (M0) into an anti-inflammatory phenotype (M2, CD206hi), as compared to EVs from an unprimed construct (MSC + BCP:EV) and MSC alone (MSC:EV). Further, we compared the potential of different EV groups in modulating the macrophage polarization response, and found that MSCp + BCP:EVs also have an enhanced potential for bi-directional macrophage polarization switching (i.e., from pro- to anti-inflammatory states and vice-versa).

In the present study, the MSC cultured with BCP secreted EVs containing substantially fewer pro-inflammatory cytokines (notably IL8, IL17 and TNFα) than the MSC cultured alone. However, this effect was changed when MSCs were pre-primed with inflammatory cytokines (MSCp + BCP) resulting in significantly more TNFα, and slightly more IL1β. Further, anti-inflammatory cytokines, (IL10 and IL5) were increased in MSC + BCP and MSCp + BCP derived EVs. In contrast, a previous study showed decreased IL10 and IL5 from inflammatory primed MSC-derived EVs (MSC EV^+^) compared to those that were unprimed [20]. Thus, the differential anti-inflammatory cytokine results in the present study could be due to BCP-mediated alterations of MSC-derived EV. In the context of biomaterial alone, it is known that biomaterial properties— such as stiffness, topography, microstructure, dimensionality, etc.—effects the overall immunomodulatory response of MSCs [35]. Specifically in case of BCP, MSCs cultured with rough BCP (nano/micro hybrid structure), were shown to produce fewer pro-inflammatory cytokines (IL6 and MMP3) and gene expression, as compared to those cultured on smooth BCP, in vitro [36]. Additionally, we also showed that several chemokines (MCP1/CCL2, MIP1b/CCL4, IP10/CXCL10 and RANTES/CCL5) essential in the monocyte–macrophage functional response during bone remodeling functions [30] were found in higher amounts in MSCp + BCP:EV. These results are in agreement with MSC EV^+^ cytokine analysis from the study mentioned above [20], suggesting a similar response due to priming, irrespective of BCP. Notably, RANTES, a prime chemoattractant for osteoblasts [37], was found to be increased only in the EVs derived by MSC + BCP groups, and not MSCs alone. Remarkably, the canonical increase in angiogenic growth factor (VEGF) seen in MSCp + BCP derived EVs suggests that they possess an enhanced neovascularization capacity, which is necessary for successful bone regeneration. A previous study has shown that rat MSC-derived EVs are capable of promoting the proliferation of endothelial cells (HUVECS) in vitro and also of enhancing bone regeneration in vivo in a subcutaneous model [38]. Overall, our results from EV cytokine analysis suggest that both the priming of MSCs and combination with BCP might have individual effects on the content of EVs produced by them. However, the presence of both factors together is required to essentially modulate the cytokine content of resulting EV cargos towards being more pro-bone-regenerative in nature. Given that most of the previous reports on the cargo analysis of MSC-derived EVs were focused on the miRNA/mRNA content [39] or proteomic analysis (with few cytokines) [40], our findings shed new light mainly on bioactive EV encapsulated cytokines as a potent source of MSC-immune cell cross-talk in tissue engineered therapy.

A growing number of studies have reported the importance of MSC–macrophage interactions (M2 polarization) in mediating early inflammation and wound healing [34]. Prevalent macrophage subset nomenclature is based on how they generate specific CD4 T cell responses i.e., the M1-like subset generates a CD4 T-helper 1 (Th1) response while the M2-like subset induces a response from a CD4 T-helper 2 (Th2) [41]. Hence, macrophage polarization is referred to as the transition between two ends of a dynamic spectrum, allowing passage through a transitional macrophage population containing mixed M1 and M2 subsets. Diverse approaches were used in the past to enhance the M2 macrophage-mediated healing response by MSC-EVs, most commonly hypoxic pre-conditioning [19,42], inflammatory priming via IFNγ alone [21], or with TNFα+IFNγ [20]. Moreover, it was reported that EVs, particularly from TNFα+IFNγ primed MSC, showed enhanced potential to attenuate inflammation [40,43]. In our study, EVs primed with TNFα+IL1β (MSCp + BCP) were employed, which showed higher levels of TNFα and IL1β (in EV cargo), thereby demonstrating that active soluble cytokines supplied artificially induced MSCs to produce more of the same cytokines, which were at least partly encapsulated within the EV compartment. Consequently, macrophages treated with these primed EVs showed enhanced switching of M0 and M1 macrophages towards the M2 subtype. This is discussed more in succeeding sections.

We found that naïve (M0) macrophages treated with MSCp + BCP:EV (primed) expressed the highest amounts of CD206 and CD163 (M2 markers) and the lowest amounts of CD80 and CD86 (M1 markers). This suggests that in the absence of M1 or M2 inducing factors, the EV cargo of MSCp + BCP constructs possess an enhanced tendency to shift naïve M0 macrophages into anti-inflammatory M2-like macrophages. Similarly, previous studies have shown that β-TCP (a major constituent of BCP) extract or hyaluronic acid/HA encapsulation (also a constituent of BCP) could elicit the macrophage response of MSCs by decreasing M1 macrophages and enhancing M2 [8]. However, those soluble factors could have originated from both CM and EV compartments, a point which was not clarified in the study mentioned above. Our results provide evidence that EVs derived from MSC + BCP constructs are at least partly involved in eliciting the M2 macrophage switching. Additionally, in agreement with our results from inflammatory primed EVs, hypoxic primed EVs from an MSC culture were shown to enhance M2 macrophage switching (compared to the normoxic MSC EV) both in vitro and in vivo using a mouse muscle regeneration model [19]. Further, at the transcriptional level, MSCp + BCP:EV-treated macrophages expressed higher M2 markers and lower M1 markers as compared to macrophages treated with MSC:EV and MSC + BCP:EV. Interestingly, the trend of mRNA expression of some of the cytokine genes analyzed (IL1β, TNFα, IL10, MCP-1, VEGFA), after EV treatment (in macrophages) across different groups, was found to be similar to the protein levels of these cytokines as measured in EVs via cytokine analysis. This further suggests that the cytokine content of EVs from different MSC groups contributed to the differential modulation of the macrophage response.

The balance of the two macrophage phenotypes (M1 and M2) has long been suggested to be critical in biomaterial-mediated apoptotic cell clearance, tissue repair mechanisms [44] and, more recently, in the osteogenic differentiation of MSCs [45]. In this regard, MSCs are well known to mitigate M1 macrophages by inducing preferential switching to M2 macrophages in a sequential manner [46] and via secretory factors [47]. Further, in the specific context of the MSC-EV-mediated macrophage response, our study showed that MSC-EVs participate in maintaining this crucial M1/M2 balance by their enhanced potency to switch both (or bi-directionally) M1–M2 and M2–M1. We demonstrated that the MSCp + BCP group showed increased potential for an M1-M2 polarization switch, while those from MSC alone and from unprimed MSC + BCP were comparable. Hence, inflammatory priming (or injury-like response) appeared to be a necessary factor for MSC-derived EVs to mediate M2 macrophage switching. In agreement with our results, other studies showed that MSC-EVs are indeed capable of inducing M1 to M2 macrophage switch in various types of tissue regeneration models, e.g., bone, muscle, cardiac and cartilage regeneration [34,48,49].

In contrast to the more commonly evaluated roles of MSC-M2 macrophage interactions in tissue healing, previous studies have also evaluated the impact of M1 macrophages in MSC-mediated bone regeneration. It was shown, in rodent bone defect models, that transplanted MSCs actually allow local dominance of M1 macrophages, leading to successful bone regeneration [50,51]. In line with these findings, we also evaluated the ability of MSC-derived EVs in regulating M2–M1 polarization switch. Our results showed significantly higher M1/M2 ratios in MSCp + BCP:EV-treated macrophages. Thus, we found that primed EVs possess a unique ability to mediate bi-directional macrophage polarization, regulating a wide variety of microenvironments from initial inflammation to healing, and then tissue repair. In contrast, lesser M1/M2 ratios were found in MSC + BCP:EV-treated macrophages, implying that independent of priming response, EVs from the MSC + BCP group in fact helped M2 macrophages to sustain their phenotype and resist switching back to an M1 phenotype. This observation is also supported by our results from EV cytokine analysis, where the MSC + BCP:EV group showed significantly fewer proinflammatory cytokines (IL8 and TNFα) and less than detectable IL1β. Additionally, the decreased uptake of MSC + BCP:EV by macrophages could also partially contribute to overall reduced macrophage polarization. We postulate that via this ability, EVs derived from MSC + BCP constructs could help prolong the anti-inflammatory (pro-healing) states during clinical treatment.

Recently, Dabrowska et al. [34] summarized the role of MSC-derived EVs (MSC-EVs) in mediating intercellular communication in several inflammatory diseases, particularly in bone- and wound-healing mechanisms. Preclinically, the transplantation of bone-marrow-derived MSC-EVs (exosomes) were also shown to promote osteogenesis and angiogenesis in femoral bone fracture models [32,52]. To this end, even though a sizeable number of studies support the direct use of MSC-EVs based therapeutics, a substantial challenge still remains in standardizing their isolation and storage conditions for clinical use [53,54]. In our study, we used size exclusion chromatography (SEC) as a method to isolate pure EVs with high integrity from MSC-conditioned media [55,56]. Although the current most commonly used technique for EV isolation is ultracentrifugation or differential ultracentrifugation (UC) [57,58], it has several disadvantages, which include the disruption of EV particles, low EV recovery, and co-precipitation of other soluble proteins as impurities. All of these properties may cause UC-derived EVs to have reduced purity. Hence, we optimized SEC to evaluate clinically relevant EV preparations (MSC + BCP constructs), considering its easy scalability for further usage with different sample volumes [59].

In using macrophage as models of in vitro inflammation and healing processes, it is important to highlight that most of the previous studies that evaluated MSC–macrophage interactions included either macrophages of animal origin (most commonly bone-marrow-derived or murine cell lines from mice, e.g., RAW 264.7 and J774A.1 [60], or human macrophage cell lines, e.g., U937 and THP-1 [61]). However, all human macrophage cell lines being tumor-derived (transformed cells), and macrophages of animal origin may not accurately represent the M1/M2 switch that is prevalent in healthy human macrophages. Additionally, cell-line-derived macrophages were shown to have altered cytokine secretion profiles (e.g., IL10) when compared with primary macrophages [62]. The present study utilized peripheral blood-derived macrophages from a healthy human donor, thus allowing evaluation of a physiologically relevant model (EV-mediated) of inflammation and healing mechanisms occurring after MSC transplantation.

One of the limitations of the current study is excluding EVs from the MSC primed group (without BCP). These were primarily excluded to retain the focus on mimicking a clinical bone regeneration model, where MSCs and BCP are being transplanted in the bone defects, and injury (due to surgery), represent the activation by pro-inflammatory cytokines. Secondly, the challenge of maintaining high numbers of primary macrophages during in vitro culture from the same buffy coat donor also explains our choice to limit the number of experimental groups. Further, as macrophages are a highly heterogeneous cell type representing many dynamic or mixed phenotypes, our study was limited by the number of markers used to characterize various macrophage subtypes. However, we employed the most commonly described macrophage surface markers for M1/M2 populations [63,64].

To further understand MSC–macrophage interactions, we hypothesize that the role of an intermediate or mixed macrophage population (M1+M2) could be critical in maintaining the M1/M2 balance and dynamics between these two dominant subtypes. We also postulate the need to evaluate the effects of MSC-derived EVs on other immune cell types, for example, neutrophils and T cell subsets, which have been suggested to mediate bone healing and regenerative outcomes [65]. Lastly, even though the current clinical translation of MSC-derived EVs poses a variety of challenges, including optimal cell source, dosing, and scalability [25], biomaterials functionalized with EVs potentially offer a favorable mode of controlled delivery for bioactive MSC secretome.

## 5. Conclusions

In the present study, we isolated and characterized EVs derived from inflammatory primed and unprimed MSC + BCP constructs. We showed that EVs derived from primed MSC + BCP constructs possess an enhanced capacity to modulate both naïve (M0) and pro-inflammatory (M1) human macrophage subsets towards an anti-inflammatory or pro-healing (M2) type. These EVs also showed a greater potential to bi-directionally switch macrophage polarization states. Such properties were attributed to the higher levels of immunomodulatory cytokines present in the MSCp + BCP derived EVs. Thus, our study suggests the potential use of biomaterial-induced MSC-EVs for the controlled release of bioactive secretome in future clinical bone regenerative therapies.

## Figures and Tables

**Table 1:** Human 27-plex cytokine screening panel.

Abbreviation	Cytokine
bFGF	Basic fibroblast growth factor
Eotaxin/CCL11	C-C chemokine 11
GCSF	Granulocyte colony stimulating factor
GMCSF	Granulocyte-macrophage colony-stimulating factor
IFNγ	Interferon-γ
IL1b	Interleukin-1β
IL1ra	Interleukin 1 receptor antagonist
IL2	Interleukin-2
IL4	Interleukin-4
IL5	Interleukin-5
IL6	Interleukin-6
IL7	Interleukin-7
IL8	Interleukin-8
IL9	Interleukin-9
IL10	Interleukin-10
IL12 (p70)	Interleukin-12
IL13	Interleukin-13
IL15	Interleukin-15
IL17A	Interleukin-17
IP10/CXCL10	Interferon gamma-induced protein 10/CXC chemokine 10
MCP1/CCL2	Monocyte Chemoattractant Protein-1
MIP1α/CCL3	Macrophage inflammatory protein
MIP1β/CCL4	Macrophage inflammatory protein
PDGFBB	Platelet-derived growth factor-BB
RANTES/CCL5	Regulated on activation, normal T cell expressed and secreted
TNFα	Tumour Necrosis Factor-α
VEGF	Vascular endothelial growth factor

**Table 2:** List of Taqman genes used in qPCR expression analysis.

Gene ID	Gene Name	TaqMan^®^ Assay ID	Amplicon Length
GAPDH	Glyceraldehyde 3-Phosphate Dehydrogenase	Hs99999905_m1	122
IL12A	Interleukin 12A	Hs00168405_m1	67
TNFα	Tumor Necrosis Factor alpha	Hs00174128_m1	80
IL1β	Interleukin 1 beta	Hs01555410_m1	91
CD206/MRC1	Mannose Receptor, C type 1	Hs00168405_m1	82
IL10	Interleukin 10	Hs00961622_m1	74
TGFβ1	Transforming Growth Factor, beta 1	Hs00998133_m1	57
MCP1R/CCR2	Monocyte Chemoattractant Protein 1 Receptor	Hs00704702_s1	61
IDO	Indoleamine 2,3-Dioxygenase 1	Hs00984148_m1	66
VEGFA	Vascular Endothelial Growth Factor A	Hs00900055_m1	59
IL6	Interleukin 6	Hs00985641_m1	89

**Table 3:** Observed normalized cytokine concentrations in range for all MSC-derived EV groups. Concentrations (pg/mL) were normalized to total protein amount (µg/mL). *n* = 2; ‘-‘ represents less than detectable concentration in range; SD—standard deviation.

Cytokine Concentrations: (pg/µg)	MSC:EV		MSC + BCP:EV		MSCp + BCP:EV	
	Mean	SD	Mean	SD	Mean	SD
Pro-inflammatory cytokines						
IL1b	0.00752	-	0.00121	-	0.02624	0.00439
IL2	0.06248	0.00126	0.00335	0.00157	0.00743	-
IL8	0.02300	0.02501	0.00666	0.00142	0.19358	0.05257
IL12(p70)	0.00208	0.00060	0.00365	0.00012	0.00425	0.00338
IL17	0.00226	0.00226	0.02007	0.00172	0.04048	0.0126
TNFα	0.88730	0.32121	0.01065	0.00877	1.28098	0.03592
IFNγ	0.01602	0.00651	0.01782	0.00559	0.03505	0.01117
Anti-inflammatory cytokines						
IL1ra	0.11891	0.00594	0.11318	0.00410	0.10508	0.03924
IL4	0.00101	0.00102	0.00027	0.00013	0.00399	0.00134
IL5	0.56833	0.52276	1.14836	0.70858	3.07281	0.69376
1L10	0.01150	0.00031	0.03547	0.00055	0.01774	0.00871
1L13	0.00112	0.00046	0.00132	0.00031	0.00183	0.00117
Wound healing and repair cytokines						
FGF basic	0.04181	-	0.02819	0.00560	0.07910	0.00835
GCSF	0.38614	0.01427	0.21457	0.02511	0.41665	0.01024
GMCSF	0.03895	0.04553	0.00755	0.00433	0.30745	0.03843
PDGFbb	0.59094	0.53008	1.24396	0.24791	1.28193	0.05983
VEGF	10.0391	2.40937	7.77386	1.8794	18.7309	0.30316
**Chemokines**						
Eotaxin	0.01515	-	0.00225	0.00054	0.02109	0.00445
IP10	0.13681	-	0.16551	0.00610	0.30435	0.10212
MCP1	0.03561	0.03995	0.00704	0.00024	0.10736	0.00844
MIP1b	0.19382	-	0.00562	0.00124	0.29157	0.04188
RANTES	0.05952	-	0.12446	0.02725	0.11663	0.02002

## Data Availability

The data presented in this study are available on request from the corresponding author.

## Supplementary Materials

The following supporting information can be downloaded at: https://www.mdpi.com/article/10.3390/cells11030470/s1: Figure S1. Generation of macrophages from CD14+ CD16- peripheral blood-derived monocytes; Table S1: Human 27-plex cytokine screening panel; Table S2: List of Taqman genes used in qPCR expression analysis; Table S3: Observed normalized cytokine concentrations in range for all MSC-derived EV groups.
