# Supplementary material for: Extracellular Vesicles Derived from Primed Mesenchymal Stromal Cells Loaded on Biphasic Calcium Phosphate Biomaterial Exhibit Enhanced Macrophage Polarization
Source: Cells. 2022 Jan 29;11(3):470. doi: 10.3390/cells11030470 (PMC8834243; doi:10.3390/cells11030470)
Supplement: Supplementary file 1 [file cells-11-00470-s001.zip › cells-1541047-supplementary.pdf]

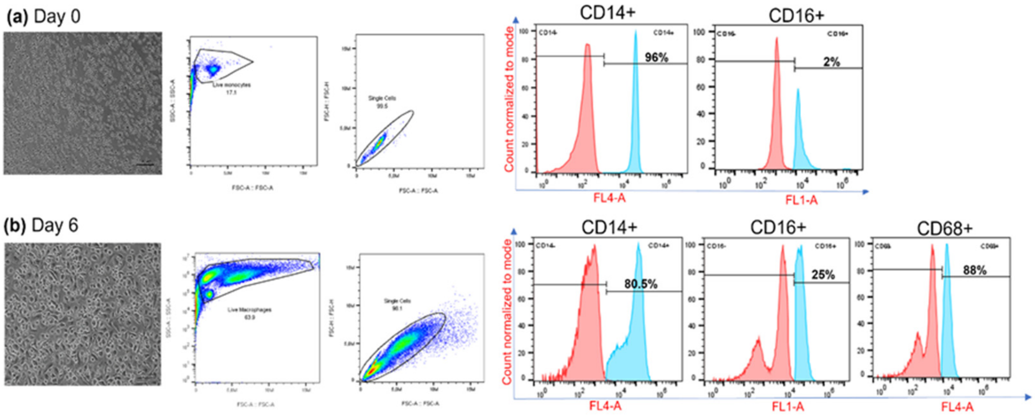

Figure S1. Generation of macrophages from CD14+ CD16- peripheral blood-derived monocytes: (**a**) freshly isolated monocytes showed 96% CD14+ and 2% CD16+ expression; (**b**) after 6 days of culture with MCSF, adherent macrophages showed around 80% CD14, 25% CD16, and additionally, 88% CD68 expression, which confirms their differentiation to M0 (naïve) state.
